# The gut microbiome is associated with behavioural task in honey bees

**DOI:** 10.1007/s00040-018-0624-9

**Published:** 2018-05-19

**Authors:** J. C. Jones, C. Fruciano, J. Marchant, F. Hildebrand, S. Forslund, P. Bork, P. Engel, W. O. H. Hughes

**Affiliations:** 1 School of Life Sciences, University of Sussex, Falmer, Brighton, BN1 9QG UK; 2 Present Address: Department of Medical Biochemistry and Microbiology, Uppsala University, Uppsala, Sweden; 3 School of Earth, Environment and Biological Sciences, Queensland University of Technology, Gardens Point, Brisbane, 4000 Australia; 4 Institut de biologie de l’Ecole normale supérieure (IBENS), Ecole normale supérieure, CNRS, INSERM, PSL Université Paris, 75005 Paris, France; 5 Structural and Computational Biology Unit, European Molecular Biology Laboratory, 69117 Heidelberg, Germany; 6 Max Delbrück Centre for Molecular Medicine, 13125 Berlin, Germany; 7 Department of Bioinformatics, University of Würzburg, 97074 Würzburg, Germany; 8 Department of Fundamental Microbiology, University of Lausanne, 1015 Lausanne, Switzerland

**Keywords:** Gut bacteria, Honey bee, Behaviour, Division of labour, Local environment, Diet

## Abstract

The gut microbiome is recognised as playing an integral role in the health and ecology of a wide variety of animal taxa. However, the relationship between social behavioural traits and the microbial community has received little attention. Honey bees are highly social and the workers perform different behavioural tasks in the colony that cause them to be exposed to different local environments. Here we examined whether the gut microbial community composition of worker honey bees is associated with the behavioural tasks they perform, and therefore also the local environment they are exposed to. We set up five observation hives, in which all workers were matched in age and observed the behaviour of marked bees in each colony over 4 days. The gut bacterial communities of bees seen performing predominantly foraging or predominantly in nest tasks were then characterised and compared based on amplicon sequencing of the 16S rRNA gene. Our results show that some core members of the unique honey bee gut bacterial community are represented in different relative abundances in bees performing different behavioural tasks. The differentially represented bacterial taxa include some thought to be important in carbohydrate metabolism and transport, and also linked to bee health. The results suggest an influence of task-related local environment exposure and diet on the honey bee gut microbial community and identify focal core taxa for further functional analyses.

**Electronic supplementary material:**

The online version of this article (10.1007/s00040-018-0624-9) contains supplementary material, which is available to authorized users.

## Introduction

The relationships between insect hosts and their microbial symbionts are increasingly recognised as being key for a variety of different ecological and evolutionary processes. Gut bacteria in particular can benefit the host by aiding nutrient acquisition, protecting against parasites and pathogens and modulating immune function and development (e.g., Koch and Schmid-Hempel 2011a; Chouaia et al. 2012; Brucker and Bordenstein 2013; Engel and Moran 2013; Engel et al. 2016). Sociality in bees especially has been previously hypothesised to be connected with gut bacterial community. Transmission between individuals in a colony is reportedly facilitated by close contact, and such transmission has been found to be important in the establishment of the honey bee and bumblebee gut microbiome (Martinson et al. 2011; Engel et al. 2012; Koch et al. 2013; Engel and Moran 2013; Powell et al. 2014; Engel et al. 2016). However, we are only beginning to understand the bidirectional relationship between the ecologically and evolutionary important behavioural and colony traits in these insects and their gut microbial communities.

In any social insect society, including bee, ant and wasp colonies, different behavioural and/or morphological castes perform different tasks, such as caring for the brood and foraging for food required for the success of the colony. These behavioural tasks are known to be associated with multiple interacting factors including age and environment, and individuals performing these different tasks are exposed to different local environments (reviewed in Oster and Wilson 1978; Beshers and Fewell 2001; Smith et al. 2008). Behavioural division of labour is perhaps epitomised by honey bees. In any honey bee colony thousands of workers forgo their own reproduction and perform the myriad of tasks required by the colony. Typically, workers performing tasks within the hive, such as feeding the brood (nurse workers), are young in age (4–12 days) (Seeley 1982) and eat a pollen-rich diet (Crailsheim et al. 1992). Foragers, on the other hand, collect resources outside the colony, including pollen and nectar, are usually older in age (approx. 15–30 days) (Seeley 1982), do not eat pollen, have low nutrient store levels and gut proteolytic enzymes, and instead are fed protein and lipids by nurses (Moritz and Crailsheim 1987; Crailsheim et al. 1992). Gut bacterial communities and behavioural phenotype in worker bees may be influenced by many of the same characteristics, e.g., diet and environment (e.g., division of labour reviewed in Johnson 2010; the bee microbiome reviewed in Engel et al. 2016). This means there is likely a link between behavioural phenotype and gut bacterial community, but to date this has been little explored (but see Kapheim et al. 2015).

Honey bees are known to harbour a consistent and unique gut bacterial community which is different to that of the solitary bees (Jeyaprakash et al. 2003; Mohr and Tebbe 2006; Cox-Foster et al. 2007; Martinson et al. 2011; Koch and Schmid-Hempel 2011b). Genomic analyses and recent functional experimental studies suggest that this core microbial community is involved in a range of key functions including nutrition and health (Engel et al. 2012; Kwong et al. 2014; Engel et al. 2014; Lee et al. 2015; Ellegaard et al. 2015; Engel et al. 2015; Raymann et al. 2017; Zheng et al. 2017; Kešnerová et al. 2017). Specifically, the gut community of worker honey bees is dominated by nine bacterial species clusters that make up 95–98% of the community (Jeyaprakash et al. 2003; Babendreier et al. 2006; Martinson et al. 2011; Moran et al. 2012; Sabree et al. 2012; Corby-Harris et al. 2014; Kwong et al. 2017). Notably similar bacterial communities have been found for workers from different populations and regions (Jeyaprakash et al. 2003; Mohr and Tebbe 2006; Cox-Foster et al. 2007; Martinson et al. 2011; Moran et al. 2012; Sabree et al. 2012). However, in a recent study we demonstrated that some dominant members of the honey bee gut bacterial community differ in relative abundance when bees are exposed to different environmental landscapes (Jones et al. 2017). Thus, there may also be differences in the gut community when bees are exposed to different local environments due to the different behavioural tasks they perform. Here we employ experimental observation colonies, in which all workers were matched in age, to examine the link between local environmental exposure driven by behavioural division of labour, and gut microbiota composition.

## Methods

### Colonies

**Fig. 1:**
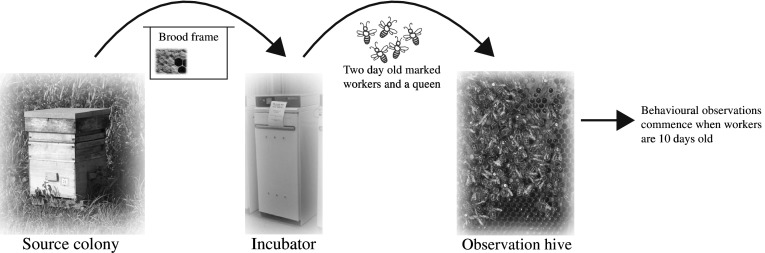
Schematic representation of experimental procedures for an exemplar colony

Five honey bee colonies were set up in two-frame observation hives at a single location near the University of Sussex in the southern UK. All colonies were maintained in a barn and each observation hive was set up with an entrance tube so workers could access the outside environment as normal. For each experimental colony, newly emerging workers were collected from a single source colony and age matched and marked individually (bees were marked using coloured number tags from Opalithplättchen, Germany). The queens of the different source colonies were unrelated and open mated. Therefore, any patterns in bacterial community seen across colonies are unlikely to be due to the genotype of the workers because each colony consisted of workers of different genotypes. Workers were matched in age to control for known age effects on bacterial community (Martinson et al. 2012). Each colony comprised approximately 1500 age matched workers, and 400 of those workers were individually marked. The workers and a queen were introduced to the observation hives when the workers were approximately 2-days-old. Observations started when the workers were 10 ± 1 days old (i.e., middle aged, Seeley 1995) (see Fig. 1 for an overview of the experimental procedures).

### Behavioural observations and sampling

The behaviour of the marked individual workers of each colony was observed and recorded for 30 min four times per day per colony for 4 days. Three major behavioural classes were observed and recorded (Seeley 1982): (1) foraging, designated as when a worker was seen leaving or returning to the hive, and with many returning foragers observed carrying pollen (as in Seeley 1982 this behavioural category may also include bees performing orientation flights); (2) food processing, when a worker was observed with her head inside a pollen or honey storage cell; and (3) nursing, when a worker was seen to put her head or whole body inside a cell containing an egg or larvae. We note that the latter two in nest tasks can in some instances also represent different elements of the single task of nursing the brood (see also Results). After the completion of all observations, all workers from each colony were shaken directly into cold absolute ethanol and all marked workers were then immediately transferred into individual tubes of absolute ethanol and stored for extraction and sequencing. Sampling was completed after dark to ensure all workers were present in the colony. Bees observed to consistently perform each behavioural task (> 75% of times observed) were selected for sequencing (see Table S1 for sampling details).

### DNA extraction, amplification and sequencing

Gut dissection and DNA extractions of individual guts was performed as outlined in Jones et al. 2017. Illumina libraries were prepared following the method outlined by Caporaso et al. 2012, by the Centre for Genomic Research, University of Liverpool. The bacterial V4 region of the 16S ribosomal gene was amplified from each DNA template in a first round PCR using the primers described by Caporaso et al. 2011, with the PCR conditions: 15 cycles of 95 °C for 20 s, 65 °C for 15 s, 70 °C for 30 s, and a final extension of 72 °C for 5 min. The amplicons were purified using Aygen SPRI Beads. A second PCR reaction was performed to incorporate Illumina adapter sequences containing indexes (i5 and i7) for sample identification. Amplicons were purified a second time, quantified on a Qubit and assessed using a Fragment Analyser. Libraries were pooled in equimolar amounts using the Qubit and Fragment Analyser data, and size selected on a Pippin prep using a range of 300–600 bps. The quantity and quality of each pool was assessed on a Bioanalyser (Agilent Technologies) and subsequently by qPCR using the Illumina Library Quantification Kit from Kapa on a Roche Light Cycler LC480011, according to manufacturer’s instructions. The amplicon pool was sequenced on an Illumina MiSeq with 15% PhiX spiked in. All sequences have been deposited in NCBI’s Sequence Read Archive and metadata for analyses are also available here (SRA PRJEB23224).

### Primary DNA processing and characterisation of microbial communities

Raw amplicon sequences were processed with the LotuS pipeline (Hildebrand et al. 2014). For this we used the command line options in LotuS: “-simBasedTaxo 2-refDB beetax” to use the Lambda aligner (Hauswedell et al. 2014) to match OTU seed sequences against a specialized reference database (see below), “-p miSeq derepMin 8:1, 4:2, 3:3” to use miSeq optimized parameters and to dereplicate only sequences occurring at least 8 times in one sample, 4 times in 2, or 3 times in 3 separate samples. For read quality filtering we used LotuS miSeq defaults, trimming reads to 220 bp and rejecting reads with an accumulated error < 1, requiring unique reads to be present at least 8 times in one sample.

In total 14,477,902 reads passed quality filtering and were subsequently clustered with UPARSE (Edgar 2013). Chimeric OTUs were filtered with uchime (Edgar et al. 2011) against a specialized reference 16S database (http://drive5.com/uchime/rdp_gold.fa). High-quality paired seed sequences for each de novo OTU were subsequently extracted, then merged with FLASH (Magoč and Salzberg 2011). These seed sequences were matched with lambda (Hauswedell et al. 2014) against a custom 16S rRNA gene database with all major known bacterial taxa associated with the honey bee gut (developed by P. Engel). For the taxonomy assignments the LotuS least common ancestor algorithm was used to assign a taxonomic identity based on the alignments to known bee taxa. OTUs were summed to genus, family, class, and phylum level per sample, according to their taxonomic classification. Additionally, we aligned all sequences against the Greengenes and Silva SSU databases using lambda (Hauswedell et al. 2014) as well as classified with RDP classifier (Wang et al. 2007). This was done to detect and then exclude any chloroplast or mitochondrial sequences to avoid their abundance confounding downstream analyses.

### Statistical analyses and comparisons of microbial communities

All downstream analyses, unless otherwise specified, were performed in R with the packages *ape, ggplot2, phyloseq, phangorn, sgof* and *vegan* (Paradis et al. 2004; Wickham 2009; Schliep 2011; McMurdie and Holmes 2013; Castro-Conde and de Uña Álvarez 2014; Oksanen et al. 2016) (code for analyses are available at https://github.com/fruciano/Material_Published_Papers/tree/master/Jones_et_al-Insectes_Sociaux). Additionally, unless otherwise specified, all the analyses were performed on samples rarefied to the smallest number of sequences per individual observed. As the results of our analyses could depend on the specific rarefied sample used, we repeated the rarefaction procedure five times. For each of the rarefied matrices, we then computed matrices of pairwise dissimilarity among individuals (Bray–Curtis, UniFrac distances). Finally, the dissimilarity matrix obtained from the first rarefied dataset was compared with each of the dissimilarity matrices obtained from the other rarefied datasets by computing their correlation and testing its significance with a Mantel test (Mantel 1967). Both the exploratory analyses and the tests of hypotheses described below were also performed on all the rarefied samples and inspected for consistency. Comparisons between rarefied samples using pairwise distances were found to be generally concordant (correlation 0.5–1; Mantel test significant in all cases). The results of the analyses were also globally consistent across different rarefactions. For these reasons, here we will report only the results based on the first rarefied sample.

To investigate patterns of microbial community diversity we computed dissimilarity matrices using Bray–Curtis dissimilarity and Unifrac distances (both weighted and unweighted). Bray–Curtis dissimilarity reflects community composition, while UniFrac distances take into account the phylogenetic relationships among members of the bacterial communities (Lozupone and Knight 2005). UniFrac distances are then either weighted by OTU abundance or unweighted (i.e., only the presence/absence of taxa/OTUs is considered). The dissimilarity matrix based on Bray–Curtis dissimilarity was used to produce exploratory ordinations using non-metric multi-dimensional scaling (nMDS) (Kruskal 1964a; Kruskal 1964b). Hypothesis testing was carried out using permutational MANOVA (PERMANOVA) (Anderson 2001). In PERMANOVA variation in distances is partitioned in terms (two factors—behaviour type and colony in our case, with colony nested in behaviour type) and tested for significance using a permutational procedure (1000 permutations). We performed PERMANOVA on all three behavioural types at the same time and compared them pairwise. In the latter case, we also verified significance after controlling for false discovery rate using the Benjamini and Hochberg procedure (Benjamini and Hochberg 1995). As PERMANOVA can suffer from lower power or higher type I error in the case of differences in dispersion between groups (Anderson and Walsh 2013), we also tested for differences in dispersion in *vegan*. This test of differences in dispersion, which is based on Anderson 2005, was performed on each of the dissimilarity measures used (Bray–Curtis, weighted UniFrac, unweighted UniFrac). We also computed the Shannon diversity index, a commonly used metric where both taxon richness and evenness of OTUs in each sample is accounted for in each individual with the “diversity” function in *vegan* and tested for differences between behaviours using ANOVA.

To test which OTUs were differentially represented between honey bees performing different tasks, we used two different procedures. First we used the procedure suggested by McMurdie and Holmes 2014 on a dataset of non-rarefied samples (excluding taxa with < 500 reads to reduce false positives due to small sample sizes). This procedure overcomes the need for rarefaction and uses the method implemented in the package DESeq2 (Love et al. 2014), which is more commonly used to detect differential gene expression in RNAseq data. The DESeq2 method fits a model based on negative binomial distribution to test for differences in gene expression (in this case read counts) between two a priori defined groups. We controlled for false discovery rate using the Benjamini and Hochberg procedure (Benjamini and Hochberg 1995). The procedure based on DESeq2 shows higher sensitivity on smaller datasets (< 20 samples per group), but tends towards a higher false discovery rate with more samples, very uneven (> 10×) library sizes or compositional effects (Weiss et al. 2017). Because of these potential limitations, we also performed an analysis of composition of microbiomes (ANCOM) (Mandal et al. 2015). This procedure has recently been found to appropriately control for false discovery rate (Weiss et al. 2017). ANCOM compares the log ratio of the abundance of each taxon to the abundance of all the remaining taxa one at a time and the Mann–Whitney *U* is then calculated on each log ratio (Mandal et al. 2015; Weiss et al. 2017). The R implementation of the procedure (version 1.1-3) was used here.

## Results

### Bacterial sequences and classification

**Fig. 2:**
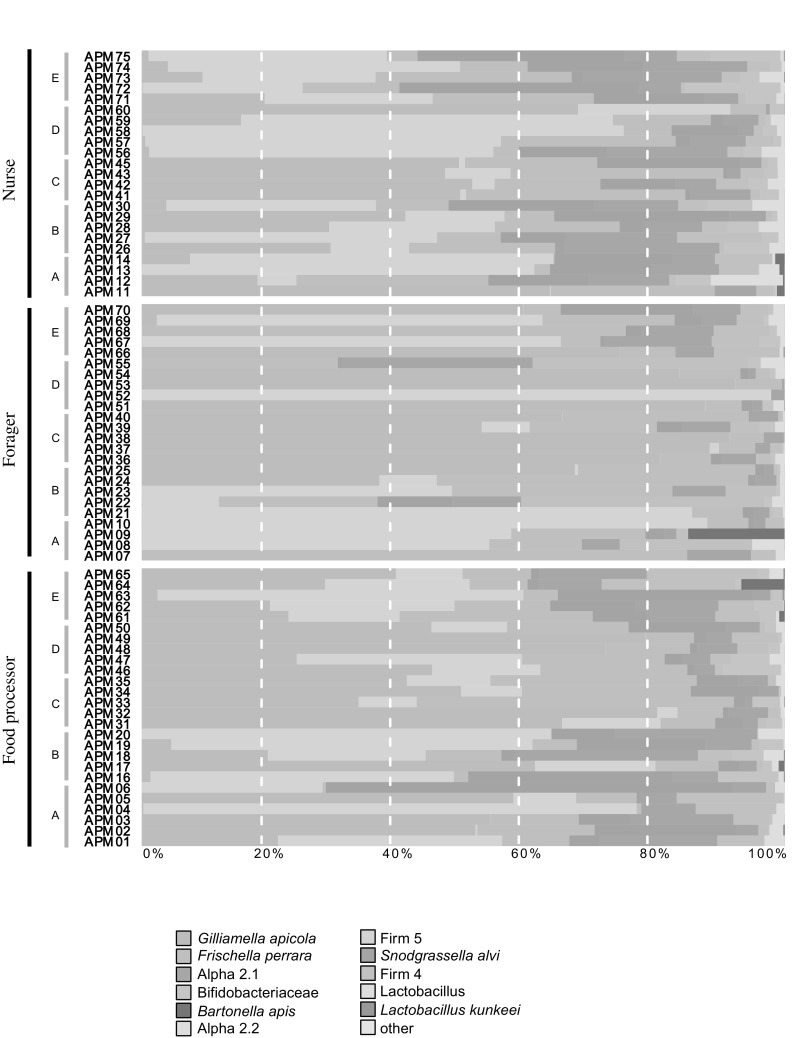
Taxonomic composition of the gut microbiome of honey bee workers performing different behavioural tasks. The proportion of each taxa in the total microbiome is represented as the proportion of the coloured bar

We obtained a total of 14,477,902 16S rRNA V4 region sequences from the 73 sampled bees assigned to the different behavioural task categories (see Table S1 for sampling details). After quality filtering, the number of sequences obtained per sample ranged from 101,846 to 344,963 reads which clustered in a total of 357 different OTUs (one sample with a low read number (35,604) was excluded for all further analyses). The main bacterial taxa previously found to dominate the gut community of honey bees were represented in high proportions in the samples studied here (Fig. 2). We were able to assign 90% of the sequence reads down to species level using a custom honey bee bacterial database, and find that the major previously identified taxa or strains were present in our data (Neisseriaceae, *Snodgrassella alvi*; Orbaceae, *Gilliamella apicola* and *Frischella perrara*; Lactobacillaceae, Firm-4 and Firm-5 species groups (genus *Lactobacillus*) and *Lactobacillus kunkeei*); *Bifidobacteriaceae; Bifidobacterium asteroides*; Bartonellaceae, *Bartonella apis*; Acetobacteraceae (Alpha 2.1 and Alpha 2.2).

### Behaviour and gut bacterial community

**Fig. 3:**
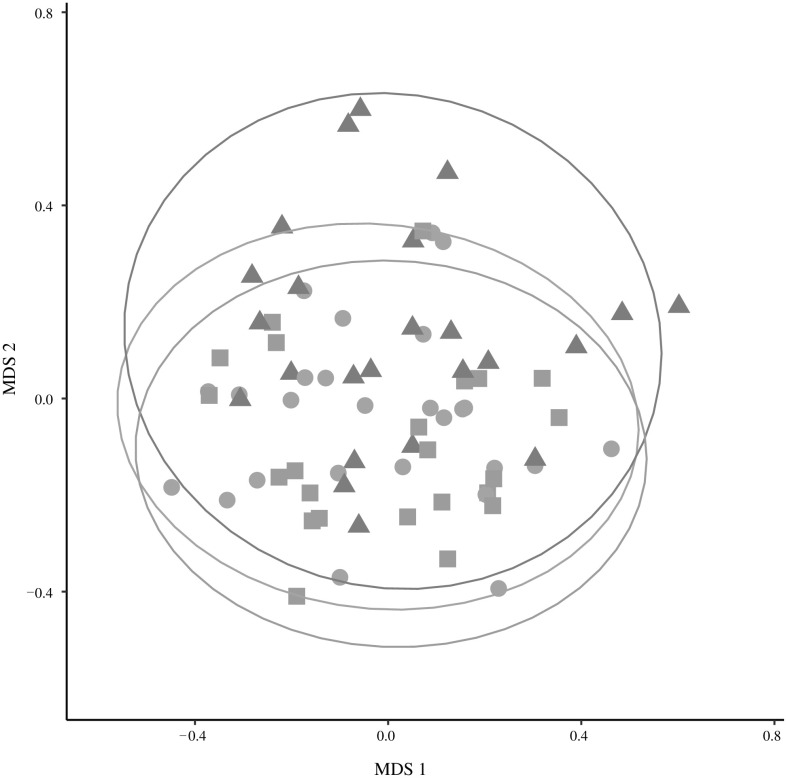
Non-metric multi-dimensional scaling plot (based on Bray–Curtis distances) of OTU frequency for the gut microbial communities of honey bees performing foraging (triangles), nursing (squares) or food processing (circles) tasks. Ellipses represent 95% confidence ellipses on the ordination

**Fig. 4:**
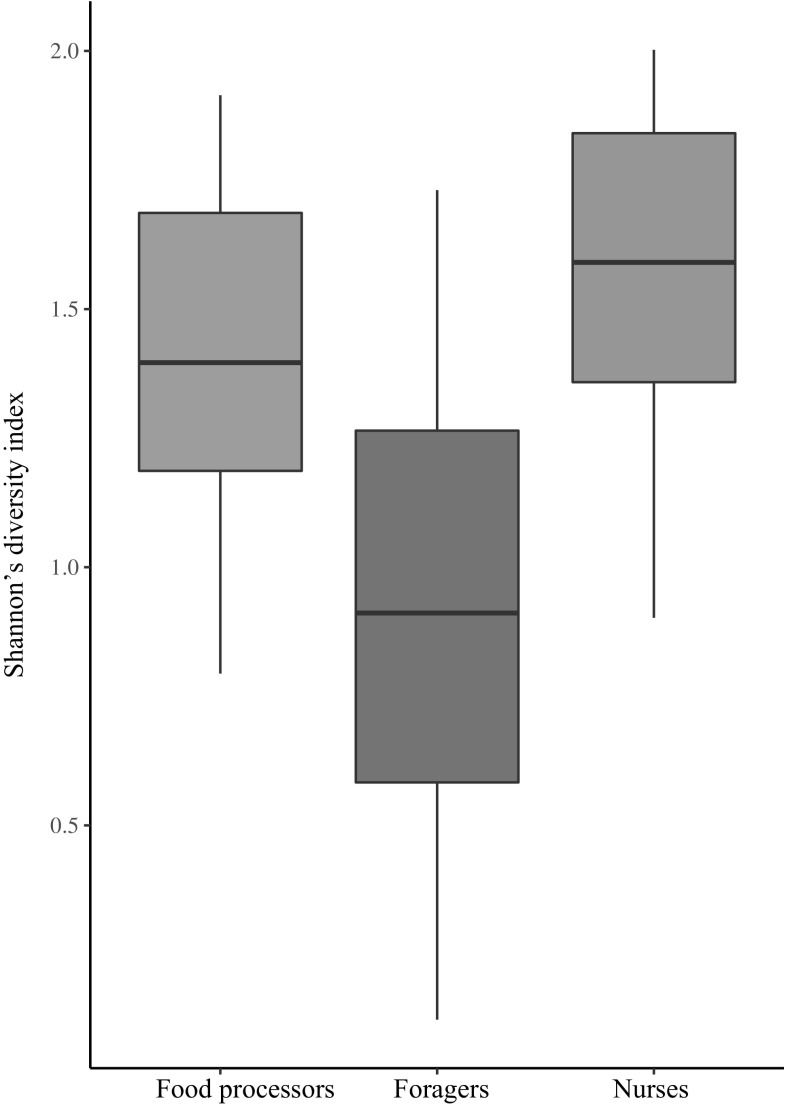
Shannon’s diversity index of OTU frequencies. Lines represent the median index value, boxes mark the interquartile range

PERMANOVA showed significant differences in gut microbial communities in honey bees performing different behavioural tasks across three different dissimilarity measures (PERMANOVA: *p* < 0.001 using Bray–Curtis dissimilarity indices, *p* = 0.001 using weighted UniFrac distances, and *p* = 0.046 using unweighted UniFrac distances). While the term for behaviour accounted for 5–12% of total variance, depending on the dissimilarity used, we also find a substantial amount of variation among colonies (Table 1). In fact variation among colonies accounted for a higher percentage of the total variance (24–26%) than variation among behaviours (Table 1). Pairwise PERMANOVA comparisons showed significant differences in the gut bacterial community of bees performing the in nest tasks compared with bees performing foraging tasks under two of the dissimilarity measures used after applying a Benjamini–Hochberg correction to each comparison (PERMANOVA, Bray–Curtis dissimilarity indices, and weighted Unifrac distances, Table S2). All pairwise comparisons using the unweighted Unifrac distances were not significant after controlling for false discovery rate. This also suggests that the value close to the significance threshold when performing PERMANOVA on all three groups with unweighted UniFrac distances (*p* = 0.046) might also be a spurious significant result. Our tests of dispersion provided support for the robustness of our PERMANOVA results to variation in dispersion among groups. In fact, PERMANOVA can lead to spurious results in the case of differences in dispersion combined with an unbalanced design (Anderson and Walsh 2013). Our three groups are only slightly unbalanced (our sample sizes for food processors, foragers and nurses are 26, 24 and 23, respectively). Even considering them as unbalanced, we do not find any significant difference in dispersion among groups using UniFrac distances (both weighted or unweighted). We also fail to find significant differences performing an ANOVA on dispersion (i.e., comparing all three groups) for Bray–Curtis dissimilarities (average distance to median: food processors 0.28, foragers 0.30, nurses 0.26; *p* = 0.055). The only case where we observe a significant difference in dispersion is in the comparison (using Tukey HSD tests) between foragers and nurses using Bray–Curtis dissimilarities (*p* = 0.043). However, in this case (variance greater in the larger group, foragers), PERMANOVA is expected to be conservative (Anderson and Walsh 2013), while we find significant differences in mean bacterial communities between these two groups. The non-metric multi-dimensional scaling (nMDS) plot shows some separation in microbial community between bees performing the behavioural task of foraging and the other two in nest tasks, but also overlap among all behavioural groups (Fig. 3). Further, gut microbiome diversity was significantly higher in bees performing the in nest tasks of nursing and food processing, compared with bees performing the task of foraging, after correction for multiple comparisons (Fig. 4, ANOVA: *F*_2,70_ = 17.64, *p* < 0.001; post hoc Tukey HSD tests, foragers versus food processors, and nurses versus foragers, *p* < 0.001).

**Table 1:** Comparison of variation in taxa/OTUs diversity among different behavioural categories and colonies (as a factor nested in behavioural category; PERMANOVA based on Bray–Curtis dissimilarity indices and UniFrac weighted and unweighted distances)

PERMANOVA	*df*	SS	MS	*F*	*R* ^2^	*p*
Bray–Curtis						
Behaviour type	2	0.36	0.18	2.45	0.06	0.001
Colony	12	1.65	0.14	1.86	0.26	0.001
Residuals	58	4.29	0.07		0.68	
Total	72	6.31			1.00	
Unifrac, unweighted						
Behaviour type	2	0.20	0.10	1.89	0.05	0.046
Colony	12	1.03	0.09	1.60	0.24	0.008
Residuals	58	3.10	0.05		0.72	
Total	72	4.33			1.00	
Unifrac, weighted						
Behaviour type	2	0.21	0.11	5.83	0.12	0.001
Colony	12	0.43	0.04	1.99	0.25	0.008
Residuals	58	1.05	0.02		0.62	
Total	72	1.69			1.00	

### Which microbial taxa differ in bees performing different tasks?

We identified which gut bacterial taxa differed between bees performing different behavioural tasks using the test implemented in DESeq2 (Table S3) and the ANCOM procedure. One of the five core members of the honey bee gut bacterial community, Firm-4 (*Lactobacillus mellis*), was found to be significantly higher in abundance in bees performing in nest tasks, both nursing and food processing, compared with bees performing the task of foraging under both ANCOM and the DESeq2-based procedure (Table S3). Taxa belonging to another core family of the honey bee gut community, Bifidobacteriaceae, were also significantly higher in abundance in bees performing in nest tasks, compared with forager bees under both test procedures. Another bacterial taxon, also belonging to the family Lactobacillaceae (Firm-5, *Lactobacillus melliventris*), was found to be significantly higher in abundance in bees performing the in nest task of nursing, compared with bees performing foraging tasks, under the DESeq2 test only. Further, bacterial taxa belonging to the phylum Proteobacteria, the recently described species *Bartonella apis* (Kešnerová et al. 2016), were found to be significantly higher in abundance in bees performing food processing tasks compared with forager bees, under the DESeq2 test only. Bacteria belonging to the Lactobacillaceae family, (*Lactobacillus kunkeei*), known to be a dominant crop (foregut) bacterium, also common in hive materials and nectar (Corby-Harris et al. 2014; Kwong and Moran 2016) were found to be higher in abundance in foragers compared with bees performing nursing behaviours. We note that very few reads were assigned to *L. kunkeei* (≪ 0.05%). Other low abundance taxa (although included in our more stringent dataset, where taxa with < 500 reads were excluded) were also found to be different in abundance between bees performing the task of foraging, compared with bees performing in nest tasks (Table S3).

## Discussion

Here we examined the association between the gut microbiome and behavioural task performance in worker bees. We show that workers matched in age, but performing different behavioural tasks and, therefore, exposed to different local environments (i.e., outside versus inside the hive) and diets, host significantly different gut microbial communities. Some members of the unique core microbial community were found to have different relative abundances in worker bees foraging for resources outside the colony, compared to workers performing in nest tasks. In addition, microbial community diversity was found to be higher in bees performing in nests tasks. Together these results, in combination with what is known to date about the functional traits of honey bee specific bacterial taxa, suggest that there may be a relationship between behavioural task, local environment exposure, and gut bacterial community.

Specifically we show that honey bee workers performing the in nest tasks of nursing and food processing have a higher relative abundance of a known core member of the honey bee bacterial community, *L. mellis* belonging to the Firm-4 species group, Phylum Firmicutes, than workers performing the task of foraging. Similarly, workers performing in nest tasks were found to have a higher relative abundance of bacteria assigned to the Bifidobacteriaceae than foragers. The latter taxa were assigned to the same family as another of the known core honey bee community taxa, the *Bifidobacterium asteroides* species cluster (Scardovi and Trovatelli 1969; Bottacini et al. 2012; Kwong and Moran 2016). Further, under the DESeq2 analysis only and, therefore, interpreted more cautiously, workers performing nursing were found to have a higher abundance of the core honey bee gut bacteria the Firm-5 species group (*L. melliventris*). Both *Lactobacillus* and Bifidobacteriaceae are thought to be associated with processing complex carbohydrates and maintaining bee health (e.g., Forsgren et al. 2010; Koch and Schmid-Hempel 2011a; Engel et al. 2012; Mattila et al. 2012; Vásquez et al. 2012; Bottacini et al. 2012; Koch and Schmid-Hempel 2012; Lee et al. 2015; Ellegaard et al. 2015; Moran 2015; Kwong and Moran 2016). In accordance with the results found here, a higher relative abundance of *Lactobacillus* species was found in non-age matched nurses versus foragers in previous work (Kapheim et al. 2015). Further, it has been suggested that lactic acid bacteria (including *Lactobacillus*) play a beneficial role in protection against pathogens in honey bees (Forsgren et al. 2010; Vásquez et al. 2012). The in nest environment may sustain a higher abundance and also diversity (as seen in the Shannon diversity results) of bacteria involved in processing carbohydrates as the hive is a large source of stored nectar and brood food, where, for example, nurse workers feed larvae food which varies in sugar content depending on the developmental stage of the larva (Brodschneider and Crailsheim 2010).

Taxa assigned to the dominant gut bacteria species *Bartonella apis* were found to be higher in abundance in bees performing the in nest task of food processing than foraging workers, under the DESeq2 test only. Interestingly in the context of the different behaviours performed by workers, *B. apis* has recently been shown to encode genes which may be involved in the degradation of secondary plant metabolites (Segers et al. 2017), and this taxon has also been found to differ in abundance depending on the landscape type the worker honey bees are exposed to (Jones et al. 2017). *L. kunkeei* on the other hand, a dominant crop (foregut) species rare in the gut, but also common in materials in the honey bee environment, was found to be higher in abundance in the gut communities of foraging workers than workers performing nursing tasks. The crop microbial environment has been suggested to be functional in inoculation and decontamination of food resources (Corby-Harris et al. 2014); however, we note that *L. kunkeei* was represented in very low read numbers. Overall we note that the differences in relative abundance of bacterial taxa between the different behavioural groups is small and direct experimental testing of the effects of these differences is required to achieve a definitive functional understanding.

Broadly, carbohydrate metabolism, and transport has been found to be the most abundant gene function category enriched in the honey bee gut microbiome in a metagenome sequencing study (Engel et al. 2012), and both *Lactobacillus* and Bifidobacteriaceae belong to taxonomic groups where carbohydrate transport and polysaccharide breakdown functions were found to be particularly abundant (Engel et al. 2012). Moreover, both groups of bacteria have recently been shown to utilize secondary plant metabolites from the outer pollen wall, such as flavonoids, phenolamides, and ω-hydroxy acids (Kešnerová et al. 2017). Potentially these bacterial taxa may be more strongly selected for in in nest workers that consume a more pollen-rich diet (Crailsheim et al. 1992), and that perform tasks within the hive such as feeding the brood. Differences in immunological and physiological functions may also play a role. Highly dominant taxa that were not found to differ in relative abundance, such as *Gilliamella apicola* and *Snodgrassella alvi*, may instead differ in the strain type associated with the different behavioural groups. Such differences cannot be detected by the community amplicon sequencing methods employed here; however, it would be interesting to extend this work to whole genome sequencing analyses in future.

It is perhaps intuitive that honey bee workers performing different tasks are exposed to different environments and consume different diets and, therefore, harbour some differences in their gut microbial community. It may also be plausible that in a feedback loop situation these differentially represented bacteria play a role in the maintenance of effective division of labour. However, future functional experiments are necessary to directly test this. Under this hypothesised scenario, environmental exposure influences bacterial community, and bacterial community in turn influences or maintains behavioural task, which in turn feeds back into environmental exposure. Bidirectional links between behaviour and bacteria have been well studied in mammals (e.g., Dillon et al. 2000; Hosokawa et al. 2008; Bravo et al. 2011; Leroy et al. 2011; Montiel-Castro 2013; Lyte 2013; Luna and Foster 2015; Stothart et al. 2016). A possible next step may therefore be to investigate the bidirectional relationship between the honey bee brain and its gut microbial community, with a focus on the bacterial taxa identified here.

Together our results show that same aged workers performing foraging versus in nest tasks differ in the relative abundance of some members of their core bacterial community. We provide insight into local environmental associations and differences in diet, and we propose a possible additional mechanism for the maintenance of behavioural division of labour. This work identifies candidate taxa for key functional investigations and underscores the complexity in the relationship between the gut microbiome, the host, and task-related environmental exposure and dietary differences.

## Electronic supplementary material

Below is the link to the electronic supplementary material.

**Table S1**. Summary of sampling design in behaviour experiment. (XLSX 30 KB)

**Table S2**. Pairwise comparisons of variation in taxa/OTUs diversity among different behavioural categories (PERMANOVA with behaviour as main factor and colony as a factor nested in behavioural category) based on Bray-Curtis dissimilarity indices and UniFrac weighted and unweighted distances. P values that remained significant after applying a Benjamini-Hochberg correction are in bold (DOCX 87 KB)

**Table S3**. Results based on pairwise comparisons of OTU representation between honey bees performing different behaviours [test implemented in DESeq2, mean = mean of normalized counts for all samples, false discovery rate controlled for using the Benjamini and Hochberg procedure (Benjamini and Hochberg <link rid="bib5">1995</link>)] (XLSX 15 KB)
