# Supplementary material for: The gut microbiome is associated with behavioural task in honey bees
Source: Insectes Soc. 2018 May 19;65(3):419–29. doi: 10.1007/s00040-018-0624-9 (PMC6061168; doi:10.1007/s00040-018-0624-9)
Supplement: Supplementary file 2 — Table S2. Pairwise comparisons of variation in taxa/OTUs diversity among different behavioural categories (PERMANOVA with behaviour as main factor and colony as a factor nested in behavioural category) based on Bray-Curtis dissimilarity indices and UniFrac weighted and unweighted distances. P values that remained significant after applying a Benjamini-Hochberg correction are in bold (DOCX 87 KB) [file 40_2018_624_MOESM2_ESM.docx]

**Table S2.**

| PERMANOVA | df | SS | MS | F | R^2^ | p |
| --- | --- | --- | --- | --- | --- | --- |
| Forager vs Nurse (Bray-curtis) | | | | | | |
| Behaviour type | 1 | 0.28 | 0.28 | 3.67 | 0.07 | **0.001** |
| Colony | 8 | 1.04 | 0.13 | 1.69 | 0.26 | 0.001 |
| Residuals | 37 | 2.84 | 0.08 |  | 0.68 |  |
| Total | 46 | 4.16 |  |  | 1.00 |  |
| Forager vs Nurse (Unifrac, unweighted) | | | | | | |
| Behaviour type | 1 | 0.08 | 0.08 | 1.36 | 0.03 | 0.209 |
| Colony | 8 | 0.55 | 0.07 | 1.19 | 0.20 | 0.204 |
| Residuals | 37 | 2.15 | 0.06 |  | 0.77 |  |
| Total | 46 | 2.78 |  |  | 1.00 |  |
| Forager vs Nurse (Unifrac, weighted) | | | | | | |
| Behaviour type | 1 | 0.20 | 0.20 | 9.38 | 0.16 | **0.001** |
| Colony | 8 | 0.24 | 0.03 | 1.41 | 0.20 | 0.163 |
| Residuals | 37 | 0.77 | 0.02 |  | 0.64 |  |
| Total | 46 | 1.21 |  |  | 1.00 |  |

| PERMANOVA | df | SS | MS | F | R^2^ | p |
| --- | --- | --- | --- | --- | --- | --- |
| Forager vs Food processor (Bray-curtis) | | | | | | |
| Behaviour type | 1 | 0.24 | 0.24 | 2.91 | 0.05 | **0.004** |
| Colony | 8 | 1.18 | 0.15 | 1.83 | 0.25 | 0.001 |
| Residuals | 40 | 3.24 | 0.08 |  | 0.70 |  |
| Total | 49 | 4.66 |  |  | 1.00 |  |
| Forager vs Food processor (Unifrac, unweighted) | | | | | | |
| Behaviour type | 1 | 0.10 | 0.10 | 1.98 | 0.04 | 0.071 |
| Colony | 8 | 0.75 | 0.09 | 1.76 | 0.25 | 0.006 |
| Residuals | 40 | 2.11 | 0.05 |  | 0.71 |  |
| Total | 49 | 2.97 |  |  | 1.00 |  |
| Forager vs Food processor (Unifrac, weighted) | | | | | | |
| Behaviour type | 1 | 0.09 | 0.09 | 4.44 | 0.08 | **0.007** |
| Colony | 8 | 0.29 | 0.04 | 1.81 | 0.25 | 0.039 |
| Residuals | 40 | 0.79 | 0.02 |  | 0.68 |  |
| Total | 49 | 1.17 |  |  | 1.00 |  |

| PERMANOVA | df | SS | MS | F | R^2^ | p |
| --- | --- | --- | --- | --- | --- | --- |
| Nurse vs Food processor (Bray-curtis) | | | | | | |
| Behaviour type | 1 | 0.05 | 0.04 | 0.68 | 0.01 | 0.810 |
| Colony | 8 | 1.13 | 0.14 | 2.11 | 0.30 | 0.001 |
| Residuals | 39 | 2.60 | 0.07 |  | 0.69 |  |
| Total | 48 | 3.77 |  |  | 1.00 |  |
| Nurse vs Food processor (Unifrac, unweighted) | | | | | | |
| Behaviour type | 1 | 0.12 | 0.12 | 2.38 | 0.04 | 0.033 |
| Colony | 8 | 0.76 | 0.10 | 1.91 | 0.27 | 0.003 |
| Residuals | 39 | 1.93 | 0.05 |  | 0.69 |  |
| Total | 48 | 2.81 |  |  | 1.00 |  |
| Nurse vs Food processor (Unifrac, weighted) | | | | | | |
| Behaviour type | 1 | 0.04 | 0.04 | 2.65 | 0.04 | 0.052 |
| Colony | 8 | 0.34 | 0.04 | 3.13 | 0.38 | 0.001 |
| Residuals | 39 | 0.53 | 0.01 |  | 0.58 |  |
| Total | 48 | 0.90 |  |  | 1.00 |  |
